# Subjective and objective cognitive function among older adults with a history of traumatic brain injury: A population-based cohort study

**DOI:** 10.1371/journal.pmed.1002246

**Published:** 2017-03-07

**Authors:** Raquel C. Gardner, Kenneth M. Langa, Kristine Yaffe

**Affiliations:** 1 Memory and Aging Center, Department of Neurology, University of California San Francisco, San Francisco, California, United States of America; 2 San Francisco Veterans Affairs Medical Center, San Francisco, California, United States of America; 3 Division of General Medicine, University of Michigan Health System, Ann Arbor, Michigan, United States of America; 4 Veterans Affairs Center for Practice Management and Outcomes Research, Ann Arbor, Michigan, United States of America; 5 Institute for Social Research, University of Michigan, Ann Arbor, Michigan, United States of America; 6 Institute of Gerontology, University of Michigan, Ann Arbor, Michigan, United States of America; 7 Institute for Healthcare Policy and Innovation, University of Michigan, Ann Arbor, Michigan, United States of America; 8 Department of Psychiatry, University of California San Francisco, San Francisco, California, United States of America; 9 Department of Epidemiology & Biostatistics, University of California San Francisco, San Francisco, California, United States of America; University of Cambridge, UNITED KINGDOM

## Abstract

**Background:**

Traumatic brain injury (TBI) is extremely common across the lifespan and is an established risk factor for dementia. The cognitive profile of the large and growing population of older adults with prior TBI who do not have a diagnosis of dementia, however, has not been well described. Our aim was to describe the cognitive profile associated with prior TBI exposure among community-dwelling older adults without dementia—an understudied but potentially vulnerable population.

**Methods and findings:**

In this population-based cohort study, we studied 984 community-dwelling older adults (age 51 y and older and their spouses) without dementia who had been randomly selected from respondents to the 2014 wave of the Health and Retirement Study to participate in a comprehensive TBI survey and who either reported no prior TBI (*n* = 737) or prior symptomatic TBI resulting in treatment in a hospital (*n =* 247). Mean time since first TBI was 38 ± 19 y. Outcomes assessed included measures of global cognitive function, verbal episodic memory, semantic fluency, and calculation as well as a measure of subjective memory (“How would you rate your memory at the present time?”). We compared outcomes between the two TBI groups using regression models adjusting for demographics, medical comorbidities, and depression. Sensitivity analyses were performed stratified by TBI severity (no TBI, TBI without loss of consciousness [LOC], and TBI with LOC). Respondents with TBI were younger (mean age 64 ± 10 y versus 68 ± 11 y), were less likely to be female, and had higher prevalence of medical comorbidities and depression than respondents without TBI. Respondents with TBI did not perform significantly differently from respondents without TBI on any measure of objective cognitive function in either raw or adjusted models (fully adjusted: global cognitive function score 15.4 versus 15.2, *p* = 0.68; verbal episodic memory score 4.4 versus 4.3, *p* = 0.79; semantic fluency score 15.7 versus 14.0, *p* = 0.21; calculation impairment 22% versus 26%, risk ratio [RR] [95% CI] = 0.86 [0.67–1.11], *p* = 0.24). Sensitivity analyses stratified by TBI severity produced similar results. TBI was associated with significantly increased risk for subjective memory impairment in models adjusted for demographics and medical comorbidities (29% versus 24%; RR [95% CI]: 1.26 [1.02–1.57], *p =* 0.036). After further adjustment for active depression, however, risk for subjective memory impairment was no longer significant (RR [95% CI]: 1.18 [0.95–1.47], *p =* 0.13). Sensitivity analyses revealed that risk of subjective memory impairment was increased only among respondents with TBI with LOC and not among those with TBI without LOC. Furthermore, the risk of subjective memory impairment was significantly greater among those with TBI with LOC versus those without TBI even after adjustment for depression (RR [95% CI]: partially adjusted, 1.38 [1.09–1.74], *p =* 0.008; fully adjusted, 1.28 [1.01–1.61], *p =* 0.039).

**Conclusions:**

In this population-based study of community-dwelling older adults without dementia, those with prior TBI with LOC were more likely to report subjective memory impairment compared to those without TBI even after adjustment for demographics, medical comorbidities, and active depression. Lack of greater objective cognitive impairment among those with versus without TBI may be due to poor sensitivity of the cognitive battery or survival bias, or may suggest that post-TBI cognitive impairment primarily affects executive function and processing speed, which were not rigorously assessed in this study. Our findings show that among community-dwelling non-demented older adults, history of TBI is common but may not preferentially impact cognitive domains of episodic memory, attention, working memory, verbal semantic fluency, or calculation.

## Abstract 2

In a population-based cohort study, Rachel Gardner and colleagues examine the associations between subjective and objective cognitive function and history of traumatic brain injury in older adults.

## Author summary

**Why was this study done?:**

- Prior studies have found that a history of traumatic brain injury (TBI) is associated with an increased risk of developing dementia later in life. It is unclear, however, whether a history of TBI has any measurable impact on cognitive function for the large and growing population of older adults without dementia who are living in the community.
- Our aim was to find out if a history of TBI is associated with measurable cognitive impairment among older adults without dementia who are living in communities across the United States.

**What did the researchers do and find?:**

- We used data collected in a large nationally representative survey study of older adults (age 51 y and older and their spouses) called the Health and Retirement Study.
- We studied 247 older adults with a history of TBI and 737 older adults with no history of TBI. We compared them on measures of subjective memory impairment (self-rated memory problems) and objective cognitive function (neuropsychological tests of attention, working memory, short-term memory, calculation, and verbal category fluency).
- We found no differences on any measures of objective cognitive function between older adults with and without a history of TBI. We did find that older adults with a history of TBI that involved loss of consciousness (LOC), but not those with a history of TBI without LOC, were 28% more likely to report subjective memory impairment compared to those with no history of TBI—even after accounting for differences in demographics, medical conditions, and depression.

**What do these findings mean?:**

- Among community-dwelling older adults across the United States, a history of TBI is not associated with any measurable impairment in the cognitive domains of attention, working memory, short-term memory, calculation, and verbal category fluency—cognitive domains that are often affected very early in Alzheimer disease. This study did not, however, rigorously evaluate the cognitive domains of processing speed and executive function—cognitive domains that have been shown to be impaired among older military veterans with a history of TBI.
- Further research is needed to determine the underlying cause of the higher rate of subjective memory impairment among older adults with TBI with LOC.

## Introduction

An estimated 2.5 million people in the US seek hospital-based care for traumatic brain injury (TBI) annually [1]. An additional 3.2 to 5.3 million are estimated to be living with TBI-related disability [2]. Lifetime prevalence of TBI is up to 40% among civilian adults [3] and is likely higher among military veterans [4]. TBI may cause immediate cognitive deficits of varying degree and duration across a variety of cognitive domains [5]. TBI is also a risk factor for dementia [6–13], suggesting that in certain vulnerable individuals TBI may contribute to progressive cognitive decline. The majority of TBI-exposed older adults, however, do not develop dementia [10,12]. Risk factors for post-TBI cognitive decline remain to be elucidated [14]. Thus, the clinical importance of a prior TBI exposure to the average community-dwelling non-demented older adult is unclear. In order to determine whether prior TBI is associated with significant cognitive deficits in this population, we need high-quality population-based studies pairing validated TBI exposure measures and detailed cognitive outcome assessments.

Most studies that have assessed detailed long-term cognitive outcomes among individuals with prior TBI have focused on clinical or convenience samples [15–17]. While these studies have greatly advanced our understanding of the long-term cognitive consequences of TBI in these specific populations, they may not be generalizable to the broader US population of community-dwelling older adults. One of the major challenges of studying the long-term consequences of prior, often remote, TBI on a population level is that it is necessary to rely upon self-report of TBI exposure, as it is usually not feasible to confirm diagnoses that may have occurred decades earlier without accessible documentation. A number of prior cohort studies of TBI and dementia have assessed lifetime TBI exposure using very brief self-report screens [18–20] that are known to miss more than 35% of people who would have endorsed prior TBI with a more comprehensive screen [21,22]. Of the few studies that have applied comprehensive TBI screens on a population level, a couple have identified a high prevalence of subjective cognitive impairment [23,24] among individuals with prior TBI. To our knowledge, however, there have been no prior population-based studies of the long-term consequences of TBI that have assessed detailed measures of objective cognitive function.

Our aim in this study was to address the knowledge gaps that have limited our understanding of the long-term cognitive consequences of prior, often extremely remote, TBI on the average community-dwelling older adult without dementia. Specifically, we sought to determine whether a history of TBI (ascertained via a comprehensive TBI screen) is an independent risk factor for not only subjective but also objective cognitive impairment in a population-based community-dwelling sample of older adults without dementia.

## Methods

### Design and protocol approval

This was a cross-sectional cohort study using publicly available secondary data from the Health and Retirement Study (HRS). The study is reported as per STROBE guidelines (S1 STROBE Checklist). All HRS respondents provided oral consent for the data used in this analysis. This study was deemed exempt by the University of California San Francisco Human Research Committee due to the use of publicly available de-identified data.

### Data source and sampling

**Fig 1:**
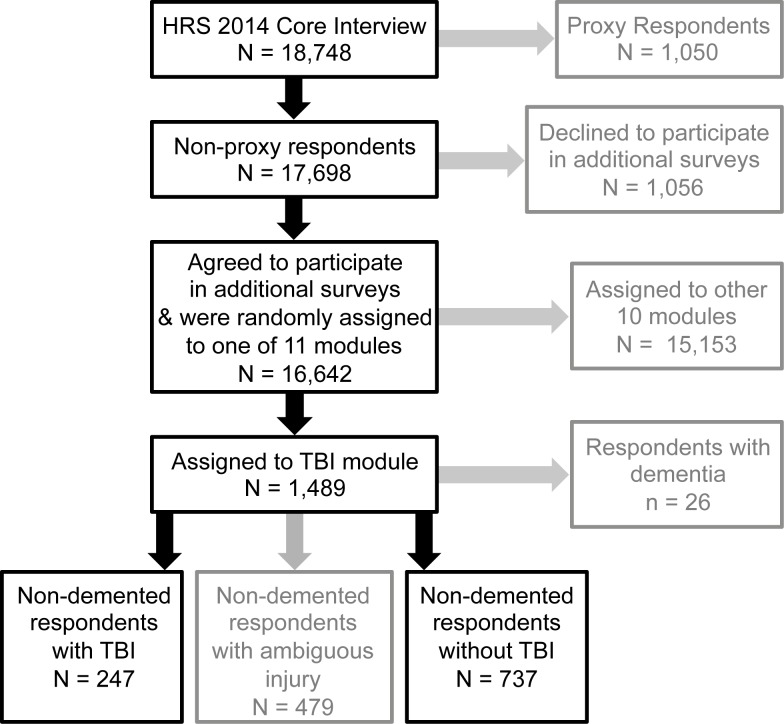
Sampling of respondents. The cohort for this study was derived from a random sub-sample of non-proxy respondents to the 2014 wave of the Health and Retirement Study (HRS) who were randomly assigned to participate in a traumatic brain injury (TBI) module. Text in grey represents respondents excluded from this study.

Data are from the HRS, a nationally representative longitudinal survey study of community-dwelling older adults (defined as adults age 51 y and older and their spouses) that began in 1992 and has continued with repeat surveys every 2 y. The HRS uses national area probability sampling of US households with supplemental oversampling of black individuals, Hispanic individuals, and Florida state residents. De-identified data are made publicly available within 1–2 y of survey completion. Detailed information about the sampling procedures, study design, instruments, and data access are available online (http://hrsonline.isr.umich.edu). For the present study, we used data from the TBI module survey that was administered to a random sub-sample of the 2014 survey respondents, as follows. The 2014 survey collected “core interview” data on 18,748 respondents. Of these, 17,698 (94%) respondents participated in the survey independently, while 1,050 (6%) respondents were unable to answer for themselves and underwent interviews by proxy respondents. Upon completion of the core interview, the 17,698 non-proxy respondents were offered participation in additional non-core surveys (“modules”). Of these, 16,642 (94%) agreed to participate in additional modules and were randomly assigned to one of 11 different survey modules. Of these 16,642 non-proxy respondents, 1,489 were randomly assigned to the TBI module (see Fig 1). Compared to the rest of the 2014 core interview non-proxy respondents, those who completed the TBI module were slightly more educated (mean [standard deviation] years education 13.6 [8.0] versus 13.2 [6.7], *p <* 0.01) but did not differ significantly on age, race, or ethnicity.

### TBI exposure

The TBI module consisted of a modified version of the Ohio State University TBI Identification Method (OSU TBI-ID). Because of its demonstrated reliability and predictive validity [25–27], the OSU TBI-ID is considered the gold-standard instrument for self-report of lifetime exposure to TBI and is recommended by the National Institute of Neurological Disorders and Stroke for use by clinical researchers studying TBI [28].

The modified OSU TBI-ID used in the HRS began with the statement “I am going to ask you about injuries to your head or neck that you may have had any time in your life.” The survey continued with six questions about injuries to the head or neck that may have been sustained in high-risk situations such as vehicle accidents and falling (e.g., “In your lifetime, have you ever injured your head or neck in a car accident…?”). If a respondent screened positive on one of these initial questions, then the survey continued with more detailed questions about the timing, associated symptoms, and need for medical attention of each reported injury up to a maximum of six injuries. The initial screening questions of the OSU TBI-ID are extremely sensitive but poorly specific as they detect injuries not only to the head but also to the neck, many of which would not fulfill clinical criteria for a TBI, which requires a traumatic impact to the head followed by neurological symptoms such as confusion, amnesia, or loss of consciousness (LOC) [29]. Furthermore, self-report of lifetime TBI, particularly early-life TBI, has been proven unreliable for TBIs that did not result in hospitalization [30]. Thus, in the present study, “TBI” was conservatively defined as any prior injury to the head or neck that (1) required treatment in a hospital (“Were you hospitalized or treated in an emergency room?”) and (2) resulted in LOC (“Were you knocked out or did you lose consciousness?”) or peri-traumatic amnesia/feeling dazed (“Were you dazed, or did you have a gap in your memory?”) or both. “No TBI” was defined as no prior head or neck injury of any kind. In order to reduce the likelihood of misclassification of TBI exposure, “ambiguous injury” was defined as any prior head or neck injury that did not meet the above criteria for TBI; respondents with ambiguous injury were excluded from subsequent analyses. For the purpose of sensitivity analyses, TBI severity (with versus without LOC) was also classified. A more precise measure of mild TBI could not be obtained because duration of LOC was not coded in number of minutes. For this reason, we chose to code severity according to LOC status rather than specific LOC duration. Although verification of TBI exposure via medical record review was not feasible in this study, our highly conservative approach to TBI exposure definition using a sensitive gold-standard instrument and exclusion of ambiguous cases makes misclassification unlikely.

### Participant selection

Of the 1,489 respondents to the TBI module, those reporting a prior diagnosis by a physician of Alzheimer disease (AD, *n =* 6) or non-AD dementia (*n =* 20) were excluded. Of the remaining 1,463 non-demented respondents to the TBI module, 479 (33%) were classified as having ambiguous injury and were excluded. The remaining 984 respondents comprised the final study cohort and consisted of 247 respondents with TBI and 737 respondents with no TBI.

### Outcomes

#### Objective cognition

Objective cognition was assessed via a global cognitive summary score and three individual items that together evaluated the domains of episodic memory, attention, working memory, verbal semantic fluency, and calculation. The HRS cognitive battery did not include detailed testing of executive function or processing speed. The HRS global cognitive summary score (short version) comprises four individual items: immediate ten-word recall (0–10 points), delayed ten-word recall (0–10 points), backward counting (0–2 points), and serial sevens (0–5 points), for a total global score of 0–27 [31]. A global score of 12 or higher is considered normal [31]. The three individual items that were assessed as unique outcomes were (1) delayed ten-word recall (0–10 points; assesses verbal episodic memory; also included in the global score), (2) animal naming in 1 min (0–unlimited; assesses semantic fluency; not included in the global score), and (3) two challenging mental calculations (defined as impaired if neither correct; assesses calculation; not included in the global score). Nearly all respondents in the study cohort completed all of the above items except for semantic fluency, which, due to idiosyncrasies of the HRS protocol, was only assessed in new respondents (not re-interviewees) age 65 y or older (*n =* 173).

#### Subjective memory

Subjective memory was measured via the following question: “How would you rate your memory at the present time? Would you say it is excellent, very good, good, fair, or poor?” Responses were coded as 1–5 with 1 being “excellent” and 5 being “poor.” Subjective memory impairment was defined as self-rated memory of less than “good” (score of 4–5).

Respondents completed the entire core HRS survey (including assessment of objective cognition and subjective memory) before they were assigned to the TBI module, thus mitigating potential expectation effects of TBI awareness on cognitive outcomes.

### Covariates

We included the following covariates that may modify or confound the association between TBI exposure and cognition: age, sex, ethnicity, race, education, self-report of physician-diagnosed medical comorbidities (hypertension, diabetes, cancer, lung disease, heart disease, stroke, smoking, and arthritis), and a measure of depression. Depression was measured via the eight-item Center for Epidemiologic Studies Depression Scale (CES-D 8), which asks respondents to report whether they experienced eight specific depressive symptoms over the past week (score 0–8). Based on prior studies, depression was defined as a score of 3 or higher [32].

### Statistical analysis

All analyses were conducted using Stata version 13 [33]. Demographics, medical comorbidities, and depression were compared between TBI and no-TBI respondents using *t*-tests for continuous variables or chi-square tests for categorical variables. Objective cognitive outcomes with scores ranging from 0 to 10 or higher (global score, verbal episodic memory, and semantic fluency) were compared between TBI and no-TBI respondents using linear regression. Ordinal categorical outcomes (calculation and subjective memory) were binarized as described above and compared between TBI and no-TBI respondents using Poisson regression with a robust variance estimator as recommended by Cummings to estimate adjusted risk ratios (RRs) for binary outcomes in Stata [34]. In order to elucidate the complex relationship between TBI, demographics, comorbidities, depression, and cognitive function, regression models were first adjusted for demographics and medical comorbidities that significantly differed between groups and were then additionally adjusted for depression (CES-D 8 score ≥ 3). To evaluate the role of TBI severity, sensitivity analyses were conducted stratified by LOC status (no TBI, TBI without LOC, TBI with LOC) and differences between TBI severity groups were assessed via tests of trend and interaction. Significance was set at *p <* 0.05.

### Analysis plan

The analysis plan was initially determined via in-person meetings of the authors in advance of obtaining the data. Once the data were obtained and cleaned, the analysis plan was modified to optimize the feasibility and scientific value of the analyses within the limitations of the available secondary data (such as missing outcome data and lack of optimally detailed coding of mild TBI exposure). Following peer review, additional minor modifications were made, including the use of an alternative HRS smoking variable with less missing data and the addition of tests of trend and interaction to the sensitivity analyses as described above.

## Results

Respondents with TBI were younger; were less likely to be female; reported higher rates of lung disease, heart disease, and arthritis; and had more depression compared to respondents without TBI (Table 1). There were minimal missing covariate data. Each individual covariate had 0% to less than 1% missing data, with the exception of the smoking covariate, which had 1.5% missing data. Overall, 2.5% of the sample was missing one or more of the Table 1 covariates, and only 0.6% of the sample was missing one or more of the covariates included in the fully adjusted model (99.4% complete caseness rate).

**Table 1:** Characteristics of respondents with and without traumatic brain injury.

Category	Characteristic	No TBI (*n =* 737)	TBI (*n =* 247)	*p-*Value
**Demographics**	**Age, years**	68.2 (11.0)	64.4 (10.1)	<0.001
	**Female**	66.3%	47.0%	<0.001
	**Hispanic**	16.4%	13.0%	0.19
	**Race**			0.98
	White	71.4%	71.7%	
	Black	20.0%	19.4%	
	Other/unknown	8.7%	8.9%	
	**Education, years**	13.2 (6.3)	13.7 (10.0)	0.32
**Comorbidities**	Hypertension	60.1%	66.0%	0.10
	Diabetes	24.7%	24.3%	0.90
	Cancer	15.9%	12.2%	0.16
	Lung disease	7.3%	13.4%	0.004
	Heart disease	18.7%	27.5%	0.003
	Stroke	5.7%	8.9%	0.08
	Ever smoker	67.5%	71.7%	0.21
	Arthritis	56.0%	68.0%	0.001
	Depression	14.1%	23.9%	<0.001

Values are mean (standard deviation) or percent.

TBI, traumatic brain injury.

The majority of respondents who reported TBI endorsed only a single prior TBI, and most TBIs involved loss of consciousness (Table 2). While average time since first TBI ranged from 0 to 98 y, the majority of TBIs occurred more than four decades ago (median 41 y). There were minimal (<3%) missing data in the outcomes assessed, except for the outcome of semantic fluency (82% missing), which was systematically missing as described above.

**Table 2:** Traumatic brain injury features.

TBI feature	Mean (SD) or percent
**Frequency**	
1	76.1%
2	21.5%
>2	2.4%
**Severity**	
Dazed/PTA only (no LOC)	28.3%
LOC	71.7%
**Timing**	
Years since first TBI	38.3 (19.0)
Years since last TBI	30.0 (19.6)

LOC, loss of consciousness; PTA, peri-traumatic amnesia; SD, standard deviation; TBI, traumatic brain injury.

Measures of objective cognitive function—global cognition, verbal episodic memory, semantic fluency, and calculation—were not significantly different between respondents with and without prior TBI in both raw and adjusted analyses (Table 3). These negative findings were unchanged in sensitivity analyses stratified by TBI severity (Table 4).

**Table 3:** Objective and subjective cognitive outcomes by traumatic brain injury status.

Outcome	Mean (SD or 95% CI) or raw or adjusted percent (95% CI)		β coefficient or RR (95% CI)	*p-*Value
	No TBI (Ref)	TBI		
**Global cognition, *n =* 958 (score 0–27)^a^**				
Raw	15.2 (4.5)	15.4 (3.9)	0.12	0.72
Model 1	15.3 (15.0 to 15.6)	15.3 (14.7 to 15.8)	−0.01 (−0.65 to 0.63)	0.97
Model 2	15.2 (14.9 to 15.5)	15.4 (14.8 to 15.9)	0.13 (−0.50 to 0.77)	0.68
**Verbal episodic memory, *n =* 972 (long-delay free recall score 0–10)^a^**				
Raw	4.3 (2.1)	4.3 (1.8)	0.01	0.95
Model 1	4.3 (4.2 to 4.5)	4.3 (4.1 to 4.6)	−0.04 (−0.33 to 0.26)	0.81
Model 2	4.3 (4.2 to 4.5)	4.4 (4.1 to 4.6)	0.04 (−0.25 to 0.33)	0.79
**Semantic fluency, *n =* 173 (number of animals named in 1 min)^a^**				
Raw	14.1 (6.4)	15.0 (5.4)	0.89	0.49
Model 1	14.0 (13.0 to 15.0)	15.7 (13.3 to 18.1)	1.70 (−0.90 to 4.31)	0.20
Model 2	14.0 (13.0 to 15.0)	15.7 (13.3 to 18.1)	1.70 (−0.96 to 4.35)	0.21
**Calculation—both items incorrect, *n =* 984^b^**				
Raw	30.0%	24.7%	0.82 (0.65 to 1.05)	0.12
Model 1	25.3% (22.9 to 27.8)	23.1% (18.6 to 27.5)	0.90 (0.70 to 1.16)	0.40
Model 2	25.5% (23.0 to 27.9)	22.4% (18.1 to 26.7)	0.86 (0.67 to 1.11)	0.24
**Subjective memory fair or poor, *n =* 984^b^**				
Raw	27.3%	34.8%	1.28 (1.04 to 1.57)	**0.021**
Model 1	23.8% (21.4 to 26.3)	29.1% (24.7 to 33.4)	1.26 (1.02 to 1.57)	**0.036**
Model 2	23.9% (21.5 to 26.3)	27.6% (23.5 to 31.7)	1.18 (0.95 to 1.47)	0.13

Statistically significant results are in bold. Model 1: adjusted for age, sex, heart disease, lung disease, and arthritis. Model 2: adjusted for age, sex, heart disease, lung disease, arthritis, and depression.

^a^Values are mean (SD) or adjusted mean (95% CI); β coefficient (95% CI).

^b^Values are raw or adjusted percent (95% CI); RR (95% CI).

RR, risk ratio; SD, standard deviation; TBI, traumatic brain injury.

**Table 4:** Objective and subjective cognitive outcomes by traumatic brain injury severity.

Outcome	No TBI (Ref)		TBI without LOC			TBI with LOC		
	Mean (SD or 95% CI) or raw or adjusted percent (95% CI)		Mean (SD or 95% CI) or raw or adjusted percent (95% CI)	β coefficient or RR (95% CI)	*p-*Value	Mean (SD or 95% CI) or raw or adjusted percent (95% CI)	β coefficient or RR (95% CI)	*p-*Value
**Global cognition, *n =* 958 (score 0–27)^a^**								
Raw	15.2 (4.5)	15.9 (3.8)		0.69	0.22	15.1 (3.9)	−0.11	0.76
Model 1	15.3 (14.9 to 15.6)	15.7 (14.6 to 16.7)		0.42 (−0.66 to 1.50)	0.45	15.0 (14.4 to 15.7)	−0.23 (−0.96 to 0.50)	0.54
Model 2	15.2 (14.9 to 15.5)	15.7 (14.7 to 16.7)		0.47 (−0.60 to 1.55)	0.39	15.2 (14.5 to 15.8)	−0.05 (−0.77 to 0.68)	0.90
**Verbal episodic memory, *n =* 972 (long-delay free recall score 0–10)^a^**								
Raw	4.3 (2.1)	4.6 (1.9)		0.23	0.38	4.3 (1.7)	−0.08	0.66
Model 1	4.3 (4.2 to 4.5)	4.5 (4.0 to 4.9)		0.11 (−0.40 to 0.62)	0.66	4.2 (3.9 to 4.5)	−0.12 (−0.46 to 0.21)	0.46
Model 2	4.3 (4.2 to 4.5)	4.5 (4.0 to 5.0)		0.14 (−0.36 to 0.65)	0.58	4.3 (4.0 to 4.6)	−0.03 (−0.37 to 0.30)	0.84
**Semantic fluency, *n =* 173 (number of animals named in 1 min)^a^**								
Raw	14.1 (6.4)	15.9 (5.0)		1.74	0.43	14.6 (5.7)	0.49	0.75
Model 1	14.0 (13.0 to 15.1)	16.4 (12.1 to 20.6)		2.29 (−2.09 to 6.66)	0.30	15.4 (12.5 to 18.3)	1.39 (−1.73 to 4.52)	0.38
Model 2	14.0 (13.0 to 15.1)	16.2 (11.9 to 20.5)		2.11 (−2.34 to 6.56)	0.35	15.4 (12.5 to 18.4)	1.43 (−1.73 to 4.59)	0.37
**Calculation—both items incorrect, *n =* 984^b^**								
Raw	30.0%	18.6%		0.62 (0.37 to 1.02)	0.06	27.1%	0.90 (0.69 to 1.18)	0.46
Model 1	25.5% (23.1 to 28.0)	18.8% (10.4 to 27.1)		0.70 (0.42 to 1.17)	0.18	24.7% (19.5 to 29.9)	0.96 (0.73 to 1.27)	0.79
Model 2	25.7% (23.2 to 28.1)	18.6% (10.3 to 26.9)		0.69 (0.42 to 1.16)	0.16	23.8% (18.9 to 28.8)	0.92 (0.70 to 1.20)	0.53
**Subjective memory fair or poor, *n =* 984^b^**								
Raw	27.3%	27.1%		1.00 (0.67 to 1.49)	0.98	37.9%	1.39 (1.11 to 1.73)	**0.004**
Model 1	23.8% (21.4 to 26.2)	23.7% (15.7 to 31.6)		1.00 (0.66 to 1.50)	0.99	31.2% (26.1 to 36.3)	1.38 (1.09 to 1.74)	**0.008**
Model 2	23.9% (21.5 to 26.3)	23.0% (15.7 to 30.4)		0.97 (0.66 to 1.44)	0.89	29.4% (24.5 to 34.3)	1.28 (1.01 to 1.61)	**0.039**

Statistically significant results are in bold. Model 1: adjusted for age, sex, heart disease, lung disease, and arthritis. Model 2: adjusted for age, sex, heart disease, lung disease, arthritis, and depression.

^a^Values are mean (SD) or adjusted mean (95% CI); β coefficient (95% CI).

^b^Values are raw or adjusted percent (95% CI); RR (95% CI).

LOC, loss of consciousness; RR, risk ratio; SD, standard deviation; TBI, traumatic brain injury.

Respondents with prior TBI were significantly more likely to endorse subjective memory impairment in both unadjusted models and models adjusted for demographics and medical comorbidities (35% versus 27%; RR [95% CI]: raw, 1.28 [1.04–1.57], *p =* 0.021; partially adjusted, 1.26 [1.02–1.57], *p =* 0.036; Table 3). Following further adjustment for depression, however, risk for subjective memory impairment was no longer significant (RR [95% CI]: fully adjusted, 1.18 [0.95–1.47], *p =* 0.13; Table 3).

In sensitivity analyses stratified by TBI severity, risk for subjective memory impairment was significantly elevated among respondents with TBI with LOC but not among respondents with TBI without LOC in all raw and adjusted models (Table 4). Adjustment for depression reduced, but did not attenuate, the risk of subjective memory impairment among those with TBI with LOC (RR [95% CI]: partially adjusted, 1.38 [1.09–1.74], *p =* 0.008; fully adjusted, 1.28 [1.01–1.61], *p =* 0.039; Table 4). While differences in the risk of subjective memory impairment between the TBI with LOC group and the TBI without LOC group did not reach statistical significance (test of TBI with LOC = TBI without LOC: raw *p =* 0.13, partially adjusted *p =* 0.14, fully adjusted *p =* 0.22), there was a statistically significant trend for greater risk of subjective memory impairment across TBI severity groups (test of trend across TBI severity groups [no TBI, TBI without LOC, TBI with LOC]: raw *p =* 0.006, partially adjusted *p =* 0.013, fully adjusted *p =* 0.065).

This study was powered (at alpha = 0.05; power = 0.8) to detect differences (effect sizes) as small as 0.85 points in global cognition, 0.39 points in verbal episodic memory, 3.2 points in semantic fluency, 9.3% in prevalence of calculation impairment, and 9.1% in prevalence of subjective memory impairment between TBI groups.

## Discussion

In this population-based study of community-dwelling older adults without dementia, prior TBI with LOC—but not prior TBI without LOC—was associated with a 38% increased risk for subjective memory impairment that was partially but not entirely mediated by comorbid depression. Furthermore, prior TBI of any severity was not associated with significant objective cognitive impairment in the domains of episodic memory, attention, working memory, verbal semantic fluency, or calculation.

Our finding that history of TBI is associated with subjective cognitive impairment is consistent with the results of two prior population-based survey studies [23,24]. One of these studies reported that 22% of respondents with prior TBI reported subjective difficulty with memory or thinking [24]. This study did not include a comparison to respondents without TBI, an assessment of this outcome according to TBI severity, or an assessment of objective cognitive function. Additionally, the survey non-response rate in this study was quite high (63%) [24], limiting the generalizability of the findings. The other survey study, which assessed multiple self-reported outcomes in a population-based sample of adults of all ages with and without TBI, reported mixed results [23]. On one measure, risk for subjective cognitive complaints was elevated among those with TBI with LOC (adjusted prevalence ratio [95% CI]: 1.44 [1.08–1.91]) but not among those with TBI without LOC (adjusted prevalence ratio [95% CI]: 1.15 [0.77–1.71]) compared to those without TBI—which is similar to our own findings. On another measure of post-concussive symptoms, however, there was increased risk for self-reported poor memory and poor concentration among all respondents with prior TBI, even those without LOC [23]. This study did not, however, adjust for medical comorbidities or depression and also did not assess measures of objective cognitive function. Our study expands upon these prior findings by demonstrating that a history of TBI with LOC is a risk factor for subjective cognitive impairment among community-dwelling older adults without dementia but that this risk is partially mediated by comorbid depression. This more nuanced understanding of post-TBI subjective cognitive impairment is important because depression may be a long-term consequence of prior TBI [16,24,35] and because depression is a treatable condition, raising hope that this negative outcome may be partially modifiable. Furthermore, our study is the first population-based study to our knowledge to demonstrate that the subjective cognitive impairment reported by older adults with a history of TBI may not be subserved by measurable impairments in objective cognitive function.

The lack of objective cognitive impairment in individuals with prior TBI found in our study may be due to a variety of factors. TBI-associated cognitive impairment among older adults may primarily impact domains not rigorously covered by the HRS cognitive battery—namely, executive function and processing speed. For example, prior studies of older military veterans have identified TBI-associated impairments only on rigorous measures of processing speed (Trail Making Test Part A [36] and Wechsler Adult Intelligence Scale–Revised Digit Symbol [37]) and executive function (Trail Making Test Part B [36] and NIH-EXAMINER fluency factor and cognitive control factor scores [38])—neither of which are part of the HRS cognitive battery—and not on detailed measures of attention, working memory, episodic memory, or language [16,17]. Alternatively, it is possible that the HRS cognitive battery was not sufficiently sensitive to detect mild impairments in the domains tested. For example, subjective cognitive impairment has been associated with the development of objective cognitive impairment decades later [39]. Thus, it is possible that measurable cognitive deficits will develop in these respondents over the next several years as they are followed longitudinally. The lack of objective cognitive impairment in individuals with prior TBI may also be due to the younger age of the respondents with TBI compared to those without TBI, though our models that adjusted for age make this interpretation less likely. Survival bias may have influenced our results if this community-dwelling non-demented sample of older adults represents a resilient segment of the US population. Lastly, the lack of evidence for objective cognitive impairment among older adults with a history of TBI may suggest that, in the general population (non-clinical, non-convenience sample), a history of TBI is not a significant risk factor for cognitive impairment or dementia. Indeed, some prior epidemiological studies have failed to identify an association between TBI and dementia [40–45]. Thus, our finding may provide support for the hypotheses that prior studies that have identified an elevated risk of dementia following TBI may have been limited by recall bias due to self-reported TBI [8,46–49], reverse causation [50], misdiagnosis of post-concussive syndrome as dementia [51], or inclusion of only clinical populations (not population-based).

Of note, the cognitive domains assessed in this study mainly include domains that are typically affected very early in typical AD: episodic memory, semantic fluency, and calculation. In fact, these domains may even be impacted in the preclinical phase of AD [52,53]. Performance on tests of episodic memory, in particular, has been found to correlate with amyloid pathology among cognitively normal older adults [54]. Thus, our finding that non-demented older adults with prior TBI do not have greater impairment in episodic memory compared to their non-TBI-exposed counterparts provides support for the hypothesis that TBI-related dementia and cognitive impairment, when present, are likely not of the AD type. Indeed, as mentioned above, prior cohort studies of older military veterans have identified impairments in executive function and processing speed among veterans with a history of fairly remote TBI compared to veterans without TBI, but have failed to identify differences on tests of attention, working memory, episodic memory, or language [16,17]. Prior cohort studies comparing cognitive profiles of patients with dementia and prior TBI to patients with dementia without prior TBI have similarly identified a non-AD pattern of deficits [18,19]. And while TBI is a fairly well-established risk factor for all-cause dementia, whether TBI is a risk factor for AD in particular remains unclear [55], with many epidemiological studies reporting increased risk for AD after TBI [6,7,9,11,12,47,56] and others reporting no increased risk [20,40] (see [55] for an excellent, well-balanced review of this topic). In light of growing evidence that the purely clinical (non-biomarker/neuropathological) diagnosis of AD is frequently inaccurate [57], it is notable that nearly all prior studies reporting an association between TBI exposure and AD-type dementia have relied upon clinical or ICD-9-code-based diagnosis of AD [6,7,9,11,12,47,56], while the few studies that have included neuropathological confirmation of AD diagnosis have failed to find an association between AD neuropathology and history of TBI [19,20].

Strengths of this study include the use of population-based, nationally representative data, a detailed cognitive assessment of both subjective and objective cognitive function, a gold-standard comprehensive TBI screen, a conservative approach to TBI classification that makes misclassification unlikely, and a careful assessment of the role of comorbid depression. This study was adequately powered to detect clinically relevant effect sizes in all outcomes assessed except for the outcome of semantic fluency. Additional strengths include the focus on community-dwelling older adults without dementia—an understudied but potentially vulnerable population.

Limitations of this study include the lack of detailed measures of executive function and processing speed, the potential for recall bias on all self-reported measures, the inability to determine onset of depression relative to TBI, and the slight bias towards higher education among respondents to the TBI module compared to the rest of the non-proxy HRS respondents, suggesting that respondents in this study may have slightly greater cognitive reserve than the general older adult population in the US [58]. Assessment of multiple outcomes without adjustment for multiple comparisons raises the possibility of a false-positive association between TBI and subjective cognitive impairment. Additional limitations include substantial systematic missing data in the outcome of semantic fluency, as this outcome was given only to first-time HRS respondents over age 65 y. The semantic fluency outcome may therefore be more prone to survival bias than the other outcomes and may also have inadequate power to detect a smaller, but potentially clinically relevant, effect size. It is reassuring, however, that the results in the domain of semantic fluency are consistent with the results across all of the other objective cognitive domains assessed. Lastly, as reflected by our high proportion of cases with TBI with LOC (72% of all TBI cases), our approach to TBI classification likely led to underrepresentation of mild TBI, thus limiting generalizability, particularly of our positive findings, to those with mild TBI.

In conclusion, this study provides, to our knowledge, the first population-based evidence that while community-dwelling non-demented older adults with a history of TBI may have increased subjective cognitive impairment compared to their counterparts without TBI, they may not have measurable impairments in the cognitive domains of episodic memory, attention, working memory, verbal semantic fluency, or calculation. While this study may provide some degree of reassurance to both older adult patients with prior TBI and their providers, further population-based studies are needed that include more comprehensive multi-domain cognitive batteries as well as measures of cognitive and depressive trajectories over time. The high prevalence of TBI of sufficient severity to require medical attention (17%) identified in this population-based study highlights the critical importance of ongoing TBI prevention efforts [59]. The high prevalence of depression identified among older adults with prior TBI (24%), in combination with the role that depression may play in increasing risk for subjective cognitive impairment, highlights an opportunity for more aggressive management of depression in this population.

## Supporting information

S1 STROBE Checklist This study meets all applicable criteria in the Strengthening the Reporting of Observational Studies in Epidemiology (STROBE) checklist. (DOC)

## Abbreviations

AD: Alzheimer disease
CES-D 8: eight-item Center for Epidemiologic Studies Depression Scale
HRS: Health and Retirement Study
LOC: loss of consciousness
OSU TBI-ID: Ohio State University TBI Identification Method
RR: risk ratio
TBI: traumatic brain injury
